# *CsMT*: a robust and streamlined *CryoSPARC* workflow for cryo-EM reconstruction of microtubules

**DOI:** 10.1107/S2059798326010144

**Published:** 2026-10-14

**Authors:** Tina Alagha, Asuva Arin, Nicholas Vangos, Helena Goodey-Parfitt, Hai Nguyen Ngo, Nhat Nam Dau, Minh Hoa Nguyen, Thibault Legal, Michael A. Cianfrocco, Khanh Huy Bui

**Affiliations:** a Department of Anatomy and Cell Biology McGill University 3640 Rue University Montreal QCH3A 0C7 Canada; b Life Sciences Institute University of Michigan Ann Arbor MI48109 USA; c Faculty of Fundamental Sciences, Hue University of Medicine and Pharmacy Hue University Hue530000 Vietnam; d Department of Biological Chemistry University of Michigan Ann Arbor MI48109 USA; e Centre de Recherche en Biologie Structurale McGill University 3649 Sir William Osler Montreal QCH3G 0B1 Canada; National Center of Biotechnology, CSIC, Spain

**Keywords:** microtubules, *CsMT*, *CryoSPARC*, cryo-electron microscopy, single-particle analysis

## Abstract

Microtubules are cytoskeletal filaments that are involved in intracellular transport, cell division and motility. Despite their biological importance, determining their high-resolution structures via cryo-electron microscopy remains a significant technical challenge due to their polymorphism and pseudo-helical assembly. Current processing workflows are complex, often requiring the integration of multiple software packages and custom scripts, which creates a steep learning curve for many research groups. To address these limitations, we introduce *CsMT*, a streamlined workflow implemented entirely within the *CryoSPARC* environment and using synthetic references. *CsMT* simplifies microtubule reconstruction by utilizing a novel protofilament-pair classification approach, which effectively handles the inherent pseudo-symmetry and structural heterogeneity of microtubules with minimal manual intervention. Our workflow is versatile, and is capable of processing both undecorated and decorated microtubules while accurately determining seams and performing high-resolution refinement. We demonstrate the efficacy of this workflow by achieving 2.3 and 2.7 Å resolution reconstructions of homotypic and heterotypic maps of undecorated microtubules, matching the best-resolved microtubule structures in the field. By unifying the pipeline into a single and portable workflow, *CsMT* enhances reproducibility and accessibility, empowering more laboratories to explore the structural biology of microtubules and associated proteins, yielding new insights into their function.

## Abstract 2

*CsMT* is a robust and streamlined *CryoSPARC*-based workflow to reconstruct undecorated and decorated microtubule maps. By unifying the processing pipeline into a single user-friendly tool, it makes high-resolution microtubule research significantly more accessible and reproducible for laboratories worldwide.

## 1. Introduction

Microtubules (MTs) are dynamic and cylindrical cytoskeletal polymers of α–β tubulin heterodimers that polymerize longitudinally to form protofilaments (PFs). These structures support a wide variety of cellular functions such as cellular structure, cell division and intracellular transport. MTs are highly dynamic polymers that undergo stochastic cycles of growth and shrinkage, a process known as dynamic instability. MTs rely on a diverse array of microtubule-associated proteins (MAPs) to balance stability with disassembly. Beyond regulating polymer dynamics, MAPs govern lattice organization, the formation of higher order structures and integration with other cellular components (Akhmanova & Kapitein, 2022 ▸). High-resolution cryo-electron microscopy (cryo-EM) studies of reconstituted MTs in complex with MAPs are essential for understanding how these proteins regulate MT organization and function. However, MTs remain challenging structural targets because they adopt pseudo-helical symmetries due to a notable lattice discontinuity: the ‘seam’. Microtubule seams exhibit lateral contacts that differ from the canonical B-lattice homotypic interactions; instead, they form A-lattice heterotypic interactions that break helical equivalence and create a structurally distinct interface along the MT.

**Figure 1:**
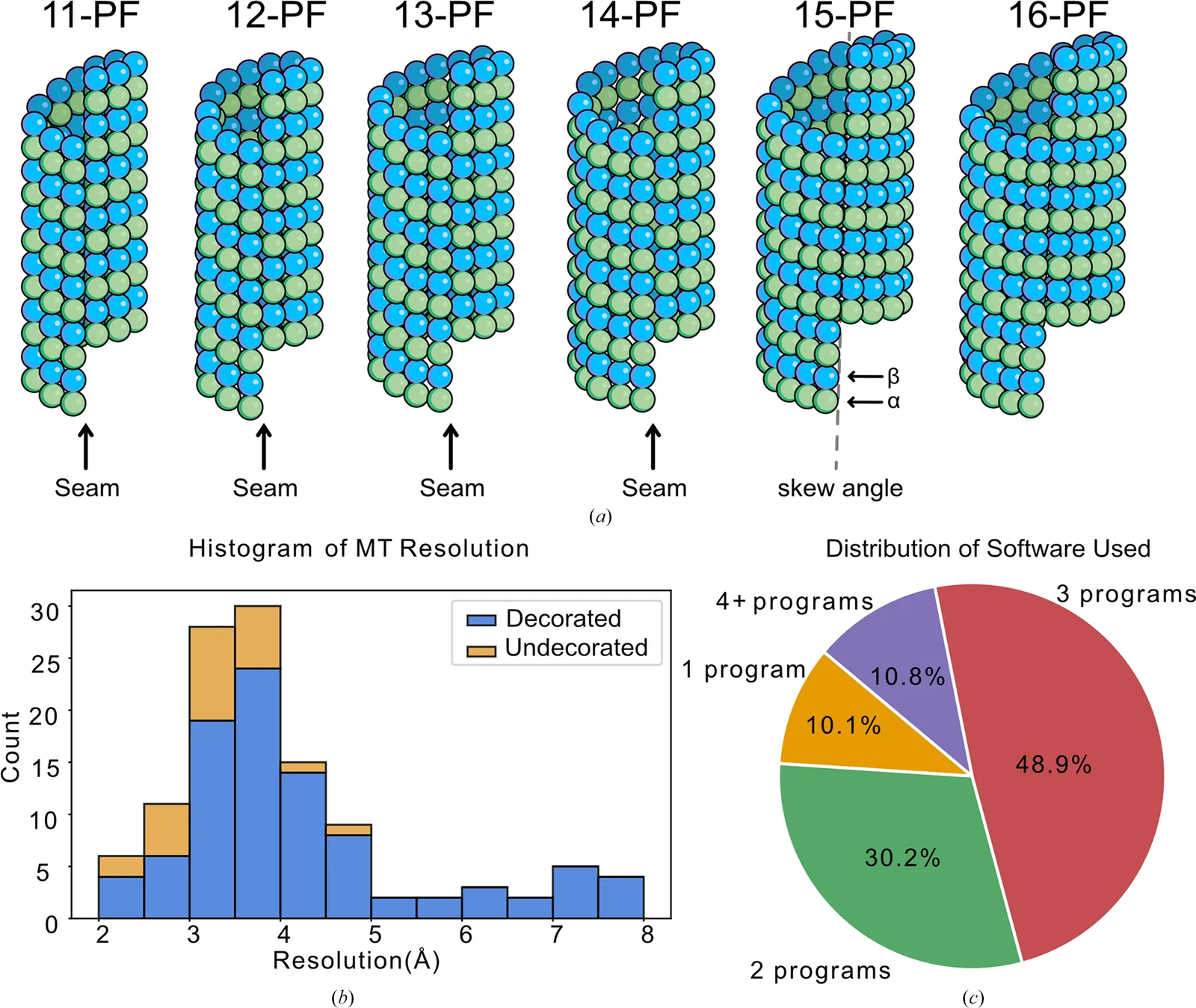
Overview of MT structures and cryo-EM reconstruction statistics. (*a*) MT polymorphism with different PF numbers. (*b*) Histogram of resolution of cryo-EM maps of decorated and undecorated MTs deposited in the Electron Microscopy Data Bank as of 13 July 2026. (*c*) Software used for MT analysis.

Identifying the seam is essential for asymmetric reconstruction and the differentiation of α- and β-tubulin. Although the two subunits are structurally similar, β-tubulin is distinguished by a longer M-loop and C-terminal tail (Nogales *et al.*, 1999 ▸). Unless the seams of the MTs are identified and aligned correctly during reconstruction, forcing symmetry onto pseudo-helical polymers can obscure such structural details (Cope *et al.*, 2010 ▸). While MTs in the cell are found to mostly contain 13 PFs, MTs ranging from nine to 16 PFs are found by reconstitution *in vitro* (Pierson *et al.*, 1978 ▸; Chrétien & Wade, 1991 ▸). The ratio of different types of MTs depends on the nucleotide and ligand used for polymerization (Sui & Downing, 2010 ▸) and can be influenced by MAPs during co-polymerization (Bechstedt & Brouhard, 2012 ▸). MTs can vary in lattice spacing, or the distance between α- and β-tubulin dimers, as GTP-bound tubulin has a 2.3 ± 0.2% wider spacing than GDP-bound tubulin (Hyman *et al.*, 1995 ▸; Alushin *et al.*, 2014 ▸). When a MT has 13 PFs, the PFs run almost parallel to the axis of the MT. Deviations from the canonical 13-PF geometry impose a tilt on the protofilaments relative to the main longitudinal axis, typically reported as a skew angle or supertwist (Fig. 1 ▸*a*). In terms of helical polymer geometry, this skew corresponds to the protofilament pitch, a parameter independent of filament diameter (Calldine, 1978 ▸). In addition, MAPs can bind at different MT interfaces such as on β-tubulin like kinesin (Kikkawa *et al.*, 1995 ▸), between four tubulin dimers like End binding protein (Zhang *et al.*, 2015 ▸) or at the seam like SPEF1 (Legal *et al.*, 2025 ▸). Therefore, MTs are inherently variable structures, differing in PF number, lattice spacing, super twisting and MAP decoration. These factors together complicate cryo-EM reconstruction workflows.

The truly helical MT forms such as 15- and 16-PF MTs can be solved by cryo-EM using classical Fourier-based helical reconstruction methods (Hirose *et al.*, 1997 ▸; Kikkawa *et al.*, 1995 ▸; Sosa *et al.*, 1997 ▸) and the iterative helical real-space reconstruction (IHRSR) approach (Egelman, 2000 ▸). With the advances in cryo-EM and the flexible nature of biological filaments, the IHRSR approach has become the standard approach to solve helical biological structure (He & Scheres, 2017 ▸). For pseudo-helical MTs, different approaches have been developed, which can be divided into two broad categories: (i) seam search-based (Sindelar & Downing, 2007 ▸; Zhang & Nogales, 2015 ▸; Zhang *et al.*, 2026 ▸; Cook *et al.*, 2020 ▸) and (ii) PF classification-based methods (Debs *et al.*, 2020 ▸; Vangos *et al.*, 2026 ▸; Zhang *et al.*, 2026 ▸; Luo *et al.*, 2026 ▸).

The principle of seam search is to find the seam based on the assumption that there is only one continuous seam along the same MT. This is achieved through three-dimensional (3D) alignment, classification, and smoothing of the φ alignment angles to find the continuous seam (Zhang & Nogales, 2015 ▸; Zhang *et al.*, 2026 ▸; Cook *et al.*, 2020 ▸). In contrast, the PF classification-based method does not assume a single seam but focuses on classification on a single or pair of PFs to distinguish the 4 nm shift classes of α- and β-tubulins (Vangos *et al.*, 2026 ▸; Debs *et al.*, 2020 ▸). In both approaches, focusing refinement on only one or two PFs rather than the entire MT (Vangos *et al.*, 2026 ▸; Zhang *et al.*, 2026 ▸; Debs *et al.*, 2020 ▸; Luo *et al.*, 2026 ▸) yields superior resolution by mitigating the effects of MT flexibility and lattice distortions introduced during vitrification. Consequently, MT structure resolution has steadily improved over the years, recently reaching beyond 2.5 Å (Fig. 1 ▸*b*). Both of these approaches work on MAP-decorated MTs but also work on undecorated MTs with some modifications (Cook *et al.*, 2020 ▸). However, structure determination of undecorated MTs is more challenging than that of decorated MTs due to the high structural similarity between α- and β-tubulins.

With these approaches, the cryo-EM workflow for MTs tends to use multiple programs and scripts such as *Frealign* (Grigorieff, 2016 ▸), *RELION* (Scheres, 2012 ▸) and *CryoSPARC* (Punjani *et al.*, 2017 ▸) (Fig. 1 ▸*c*). In particular, seam-search pipelines often involve manual picking, external scripts and particle pre-averaging (Zhang & Nogales, 2015 ▸; Zhang *et al.*, 2026 ▸; Cook *et al.*, 2020 ▸). This complexity poses a barrier for new cryo-EM users and laboratories lacking specialized expertise in this area. As a result, routine MT reconstruction remains less accessible, limiting the diversity of MAP–MT complexes and MT states that can be systematically characterized. Single-package approaches have been proposed using the two most popular single-particle cryo-EM packages: *RELION* (Vangos *et al.*, 2026 ▸) and *CryoSPARC* (Zhang *et al.*, 2026 ▸). Recently, *MiCSPARC* has been developed as a new *CryoSPARC* pipeline utilizing *CryoSPARC* in combination with external scripts for fitting, angle unification and seam search. *CryoSPARC* offers better computational optimization for large datasets. Furthermore, *CryoSPARC* has the Workflow feature, which can be generated, exported and shared between different laboratories, allowing consistent analysis and ease of use for new users. However, this functionality is incompatible with external custom scripts.

Here, we introduce *CsMT*, a PF classification-based pipeline to solve the accessibility and reproducibility of MT reconstruction. *CsMT* is a minimal *CryoSPARC*-only workflow utilizing a novel PF-pair classification approach that does not require any external software or custom scripts. *CsMT* is designed to reconstruct both decorated and undecorated MTs to high resolution. By providing a shareable and easily adjustable *CryoSPARC* workflow, *CsMT* delivers consistent, high-resolution results with minimal manual effort, making it a more reliable and efficient alternative to existing approaches.

## 2. Methods

### 2.1. EMDB map statistics

To query the MT maps in the EMDB, we used the following query: "title:"microtubule" NOT title:"doublet" AND sample_name:"tubulin" AND structure_determination_method:"singleParticle"".

The resolution histogram was obtained using a Python script with a bin of 0.5 Å resolution and a resolution cutoff of 8 Å.

For the software use statistics, the software reported in EMDB are not accurate. Therefore, we read all of the manuscripts and annotated the software manually. The plot was then plotted using a Python script.

### 2.2. Processing of the undecorated MT dataset

The dataset for Taxol-stabilized human MTs has been used previously (Vangos *et al.*, 2026 ▸).

All data processing was executed within *CryoSPARC* v.5.0.2 utilizing a cluster node equipped with two NVIDIA RTX 3090 GPUs. The raw dataset consisting of 3106 movie stacks was subjected to patch motion correction and patch CTF estimation. Micrograph curation was performed to discard low-quality images, retaining only those with a CTF fit resolution better than 6.0 Å and a defocus value below 2.5 µm.

An initial particle-picking round was performed on a representative subset of 300 micrographs using the Filament Tracer utility, configuring a characteristic filament diameter of 220 Å and a periodic unit length of 41.5 Å. This generated an initial stack of 25 608 particles, which were subjected to 2D classification into 50 classes with an initial uncertainty factor of 2. Four well resolved, representative MT classes were selected to serve as templates for comprehensive picker targeting. The comprehensive Filament Tracer run was then deployed across the curated micrograph pool utilizing the selected templates with a 220 Å diameter, 41.5 Å pitch, low/high hysteresis thresholds of 91 and 98, and a standard Gaussian blur factor of 0.2. Following an Inspect Picks step to optimize the power score and filament curvature thresholds, a total of 320 023 particles were selected. Particles were extracted using a 720-pixel box and Fourier-cropped to 180 pixels. The particle stack was cleaned via two successive rounds of 2D classification into 50 and 100 classes, with uncertainty factors set to 2 and 4, respectively, finally yielding 312 048 clean particles.

To resolve structural heterogeneity arising from MT polymorphism, the particle stack was subjected to Heterogeneous Refinement using synthetic 12-, 13-, 14- and 15-PF MTs starting volumes as reference models (Vangos *et al.*, 2026 ▸). A subset of 200 251 particles assigned to the 13-PF classes was selected and re-extracted at unbinned pixel size with a 720-pixel box. This stack was processed via Helical Refinement by enforcing 13-fold helical symmetry parameters with a rise of 9.59 Å and a twist of 27.69°, resulting in a consensus reconstruction at 3.2 Å resolution. Structural details were further improved through sequential rounds of Local CTF Refinement and Global CTF Refinement, followed by another round of helical refinement, driving the resolution to 3.0 Å. Subsequent Reference-Based Motion Correction paired with a final consensus Helical Refinement yielded a 2.9 Å resolution map. The 13-PF consensus particle stack was then symmetry-expanded based on the refined helical operators, yielding 2 600 416 symmetry-expanded units. Following a Local Refinement step that reached 2.8 Å resolution, a strict orientation filter was applied to remove all particles with a tilt angle greater than 20°. The remaining particles were prepared for focused classification by performing signal subtraction targeting either individual PFs (1-PF) or pairs of adjacent PFs (2-PF).

The 2-PF subtracted particle stack was centered using the Volume Align Tool and cropped to a 400-pixel box via Downsampling Particles. An initial Local Refinement of this stack yielded a 2.5 Å resolution reconstruction. To break the pseudo-symmetry and resolve the true 8 nm repeating registry of the α/β-tubulin heterodimers, targeted 3D classification strategies were executed. Synthetic references containing 3 × 2 tubulin dimers with a defined α/β-first starting configuration were assembled in *UCSF ChimeraX* from an *AlphaFold* model and converted to 4 Å resolved density maps using the *ChimeraX* molmap command. Custom masks for classification were built by mapping the PDB model to a 15 Å density in *ChimeraX*, followed by processing via *CryoSPARC* Volume Tools using a four-pixel dilation radius and a 12-pixel soft padding width. Using distinct PF1 and PF2 masks, two independent 3D classification tracks separated the particles into two categories, achieving an identical 51.3% to 48.7% distribution across both runs.

For the 2-PF homotypic reconstruction, particles belonging to Class 1 from both classification tracks were pooled together to form a subset of 997 466 particles. Initial volume generation was completed using Homogeneous Reconstruct Only, followed by Local Refinement that yielded a 2.5 Å resolution map. Applying a final local CTF refinement with a 500 Å search range coupled to localized refinement yielded a final homotypic map at 2.3 Å resolution. For the 2-PF heterotypic reconstruction, particles from Class 0 of the PF1 classification track and Class 1 of the PF2 classification track were combined to isolate the heterotypic assembly, resulting in 265 812 particles. This subset was processed through an identical workflow of Homogeneous Reconstruct Only followed by Local CTF Refinement and Local Refinement, resulting in a final heterotypic map at 2.7 Å resolution. For the 1-PF subtracted track, an identical signal subtraction and downsampling protocol was followed. This subset required only a single classification track to sort the tubulin registry, resulting in a final homogeneous pool of 1 275 502 particles. Final Local Refinement of this dataset produced a high-resolution map at 2.3 Å.

Final unsharpened maps were post-processed using *phenix.density_modification* (Terwilliger *et al.*, 2020 ▸) to maximize feature contrast and connectivity. Structural visualizations and figures were generated using *UCSF ChimeraX* (Meng *et al.*, 2023 ▸). To facilitate domain coloring and analysis, atomic coordinates of the tubulin PF (Vangos *et al.*, 2026 ▸) were rigid-body fitted into the 1-PF, 2-PF homotypic and 2-PF heterotypic reconstructions.

### 2.3. Processing of a decorated MT dataset

The kinesin-decorated, GMPCPP-stabilized MT dataset was obtained from EMPIAR (EMPIAR-13372; Zhang *et al.*, 2026 ▸). Similar to the undecorated MT dataset, we processed the raw dataset within *CryoSPARC* v.5.0.2 utilizing the same cluster node by tweaking some of the parameters in the workflow.

The raw dataset comprised 1676 micrographs that were subjected to motion correction and patch CTF estimation. Like the undecorated dataset, micrograph curation was performed to retain only images with a CTF fit resolution better than 6.0 Å and a defocus below 2.5 µm.

An initial particle-picking round was performed to generate templates on a representative subset of 50 micrographs using the Filament Tracer utility, configuring a characteristic filament diameter of 360 Å and a periodic unit length of 82 Å, yielding an initial stack of 664 particles.

These were subjected to 2D classification into ten classes with an initial uncertainty factor of 2, and two well resolved, representative MT classes with clear kinesin decoration were selected to serve as templates for picking across the full dataset. The comprehensive Filament Tracer run used the same 360 Å diameter and 82 Å pitch with low/high hysteresis thresholds of 91 and 98 and a Gaussian blur factor of 0.15. Following an Inspect Picks step to optimize the power score and filament curvature thresholds, a total of 47 167 particles were selected. Particles were extracted with a 938-pixel box, Fourier-cropped to 180 pixels and cleaned via two rounds of 2D classification into 100 classes, with an uncertainty factor set to 4, yielding 24 076 clean particles.

Heterogeneous Refinement was performed using the same references from 12 PF to 15 PF. The 19 888 particles assigned to the 14-PF class were selected for Extraction as this class contains 85.58% of all of the particles in the dataset. Extraction was performed with a 938-pixel box and 720-pixel Fourier-cropped box. Helical Refinement of the stack processed with a rise of 8.94 Å and a twist of 25.76° produced a consensus reconstruction at 3.6 Å resolution. Sequential rounds of Local and Global CTF Refinement followed by another Helical Refinement improved the resolution to 3.1 Å. The 14-PF particle stack was then subjected to Symmetry Expansion on the refined helical operators, yielding 553 140 symmetry-expanded particles. A subsequent Local Refinement with a full-coverage mask reached 3.0 Å, with no particle rejection, and the stack was prepared for subtraction targeting 1-PF with kinesin using a subtraction mask.

The subtracted particle stack was centered using the Volume Align Tool and cropped to a 400-pixel box via Downsampling Particles. An initial Local Refinement of this stack yielded a 2.8 Å resolution reconstruction. To define α/β-tubulin starting in the PF, 3D classification strategies were executed with two synthetic references containing 3 × 2 tubulin dimers with a defined α/β-first starting configuration assembled in *UCSF ChimeraX* from PDB entry 9t17 (Zhang *et al.*, 2026 ▸) and converted to 4 Å resolution density maps using the *ChimeraX* molmap command.

3D classification tracks separated the particles into two categories, achieving a 50.5% to 49.5% distribution. One of the classes was selected for final local refinement, resulting in 273 885 particles. Local CTF Refinement followed by a final Local Refinement was performed with an initial low-pass resolution of 8 Å using a mask covering two tubulin dimers and kinesins, producing the final map at 2.6 Å resolution.

All structural figures were prepared in *UCSF ChimeraX* (Meng *et al.*, 2023 ▸). To facilitate domain coloring and analysis, atomic coordinates of the tubulin and kinesin from Zhang *et al.* (2026 ▸) were rigid-body fitted into the 1-PF map.

## 3. Results

### 3.1. *CsMT* can reconstruct undecorated MTs using a novel PF-pair classification approach

The *CsMT* pipeline is a PF-classification approach with similarity to a method developed using *RELION* (Vangos *et al.*, 2026 ▸). For single PF reconstruction, our method is essentially identical to the method implemented in *RELION* (Vangos *et al.*, 2026 ▸). For our *CryoSPARC* workflow, we introduced two important distinctions: (i) 4 nm filament picking instead of 8 nm filament picking to prevent particle duplication during refinement and symmetry expansion caused by the structural similarity between α- and β-tubulin (Supplementary Fig. S1) and (ii) a robust ‘PF-pair classification’ logic, allowing us to reconstruct two-PF seam and non-seam structures without the need for any external scripts. A similar PF-pair classification concept was developed independently for *RELION* (Vangos *et al.*, 2026 ▸). However, isolating homotypic and heterotypic lattices still requires a Python script to calculate the intersection of the 3D classification results.

**Figure 2:**
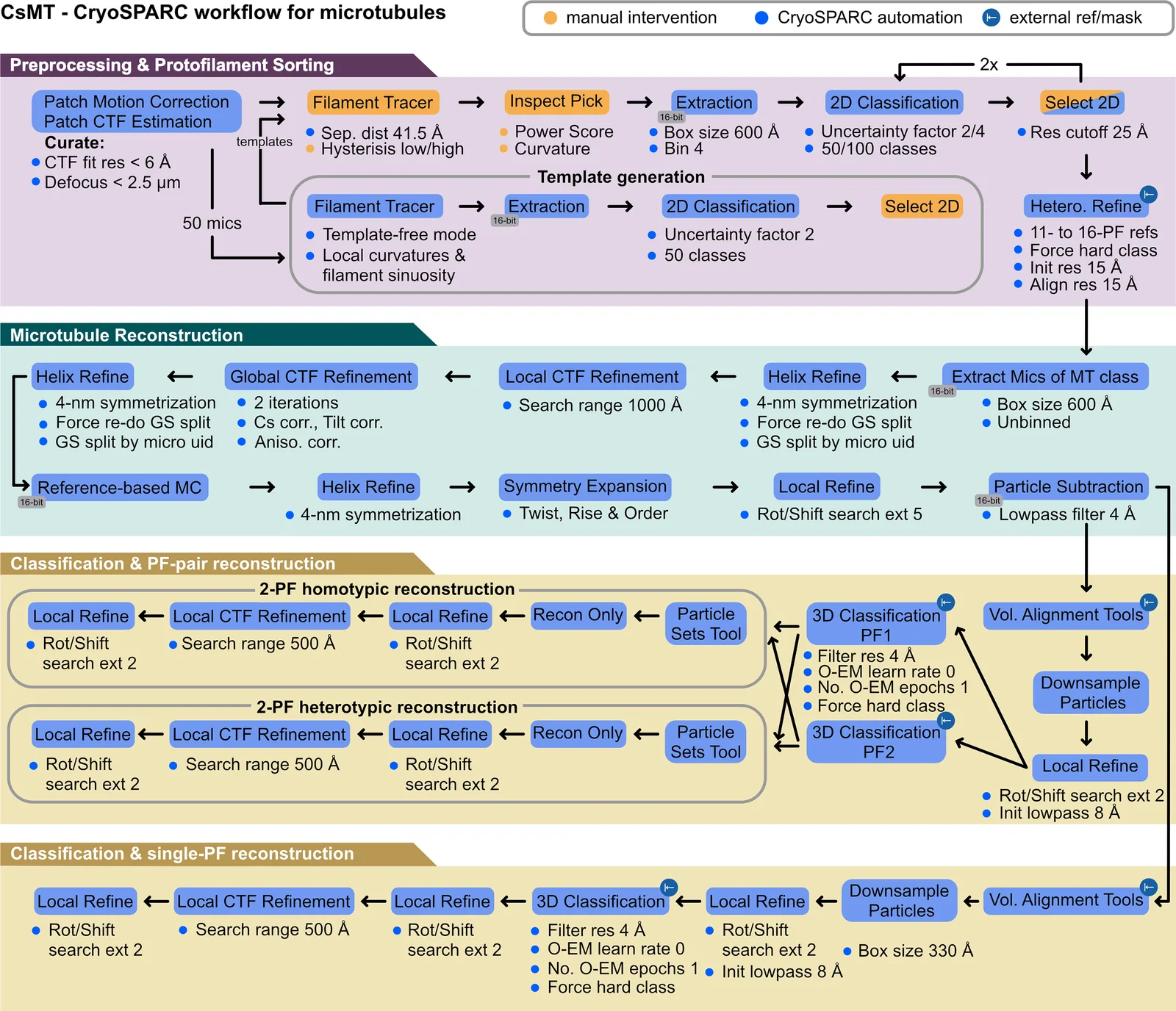
*CsMT* processing workflow.

The full workflow is detailed in Fig. 2 ▸.

#### 3.1.1. Preprocessing

Beam-induced motion and contrast transfer functions (CTF) were corrected using Patch Motion Correction and Patch CTF Estimation. Micrographs were discarded if the CTF fit resolution exceeded 6 Å or the defocus exceeded 2.5 µm.

#### 3.1.2. Filament picking

MTs were picked in two rounds using the Filament Tracer. First, a template-free picker was run on 40–100 representative micrographs, and the resulting particles were 2D-classified to generate initial templates. To ensure that rare polymorphs (for example 12-PF MTs) are captured, more micrographs should be used for this initial template generation. These templates were then deployed across the entire dataset with a 41 Å picking interval to sample both α- and β-tubulin subunits indiscriminately. Finally, two rounds of 2D classification were performed to remove false positives and damaged filaments.

#### 3.1.3. Classification and helical refinement

MT polymorphism was resolved via heterogeneous refinement in *CryoSPARC* using synthetic 11-PF to 16-PF references. While 11-PF and 16-PF structures were rare and can be omitted, including 12-PF to 15-PF references is essential for clean separation. Particles from the most abundant class (typically 13-PF or 14-PF) were selected, recentered and re-extracted unbinned. Initial helical refinement was performed, yielding a 4 nm repeat reconstruction with averaged α/β-tubulin density. To maximize resolution, helical refinements were iteratively interleaved with Local CTF Refinement, Global CTF Refinement and Reference-based Motion Correction.

#### 3.1.4. Single-PF refinement

**Figure 3:**
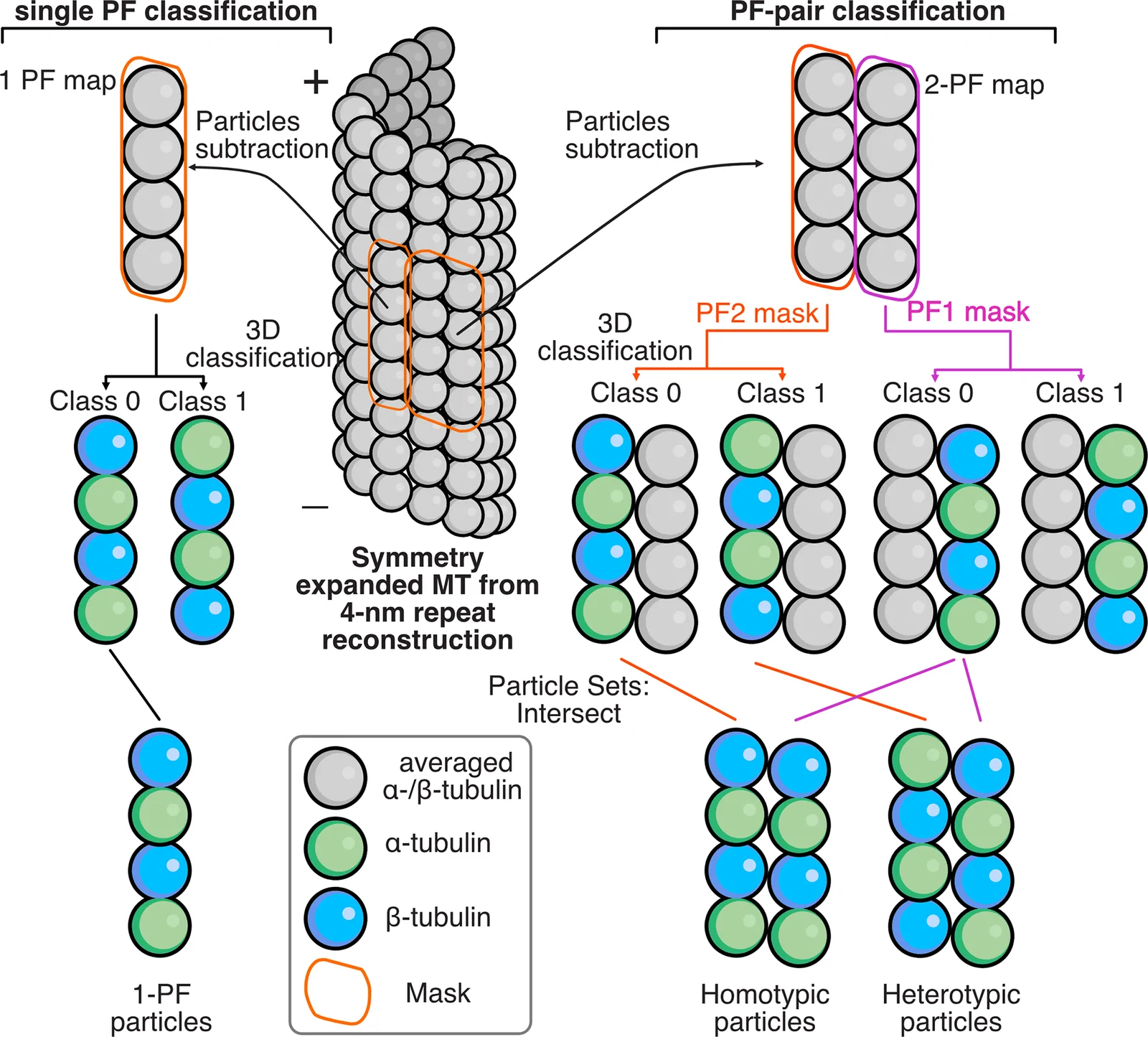
Principle of classification and grouping for 1-PF and 2-PF pair reconstruction. For clarity, a 2 × 2 tubulin dimer subtraction mask is shown here, but a 3 × 2 mask is preferred as it provides more signal for classification.

To resolve the α/β-tubulin registry, a signal subtraction mask was applied to isolate a single PF (Fig. 3 ▸). Subtracted particles were recentered, cropped and subjected to Local Refinement. Next, 3D classification with two synthetic references, representing ‘α-first’ and ‘β-first’ orientations, separated the particles into two 4 nm axially shifted classes (typically a ∼50:50 distribution). This 3D classification is consistent and reproducible because both classes have equal numbers of particles. Local refinement of either class resolves the correct 8 nm tubulin dimer repeat. Final maps were further improved by optimizing per-particle defocus values via Local CTF Refinement, followed by a final Local Refinement.

#### 3.1.5. Two-PF refinement

To visualize both homotypic (B-lattice) and heterotypic (seam) lateral interactions, a two-PF refinement approach was developed. Density corresponding to a block of 3 × 2 tubulin dimers across two adjacent PFs was isolated via signal subtraction (Fig. 3 ▸). Subtracted particles were recentered, cropped and subjected to Local Refinement. To determine the α/β-tubulin registry for each filament, focused 3D classification was performed independently for each PF using 2-PF masks containing two dimers each (PF1 and PF2) and synthetic references representing the 4 nm axial shift.

Intersecting these classification results isolated specific lattice architectures: particles belonging to discordant classes (for example class 0 for PF1 and class 1 for PF2) represented the heterotypic seam, whereas concordant classes (class 0 for both PFs) represented the homotypic B-lattice. These separated populations were then individually optimized using a cycle of Local Refinement, Local CTF Refinement and a final Local Refinement.

By applying PF-pair 3D classification with two separate PF masks, we can reproducibly separate particles into α-first and β-first classes within each PF. This approach avoids performing a 2-PF 3D classification into four classes, homotypic α-first, homotypic β-first, heterotypic αβ-first and heterotypic βα-first, using a single mask covering both PFs. Because the expected stoichiometry for these four classes is heavily imbalanced at 12:12:1:1 (assuming a 13-PF MT with one seam), single-mask classification fails to reliably isolate the rare heterotypic classes due to particle-attraction problems (Vilas *et al.*, 2022 ▸).

#### 3.1.6. Map post-processing

Final reconstructions were post-processed using *phenix.density_modification* (Terwilliger *et al.*, 2020 ▸) or *EMReady*2 (Cao *et al.*, 2026 ▸) to improve interpretability for downstream atomic modeling.

**Figure 4:**
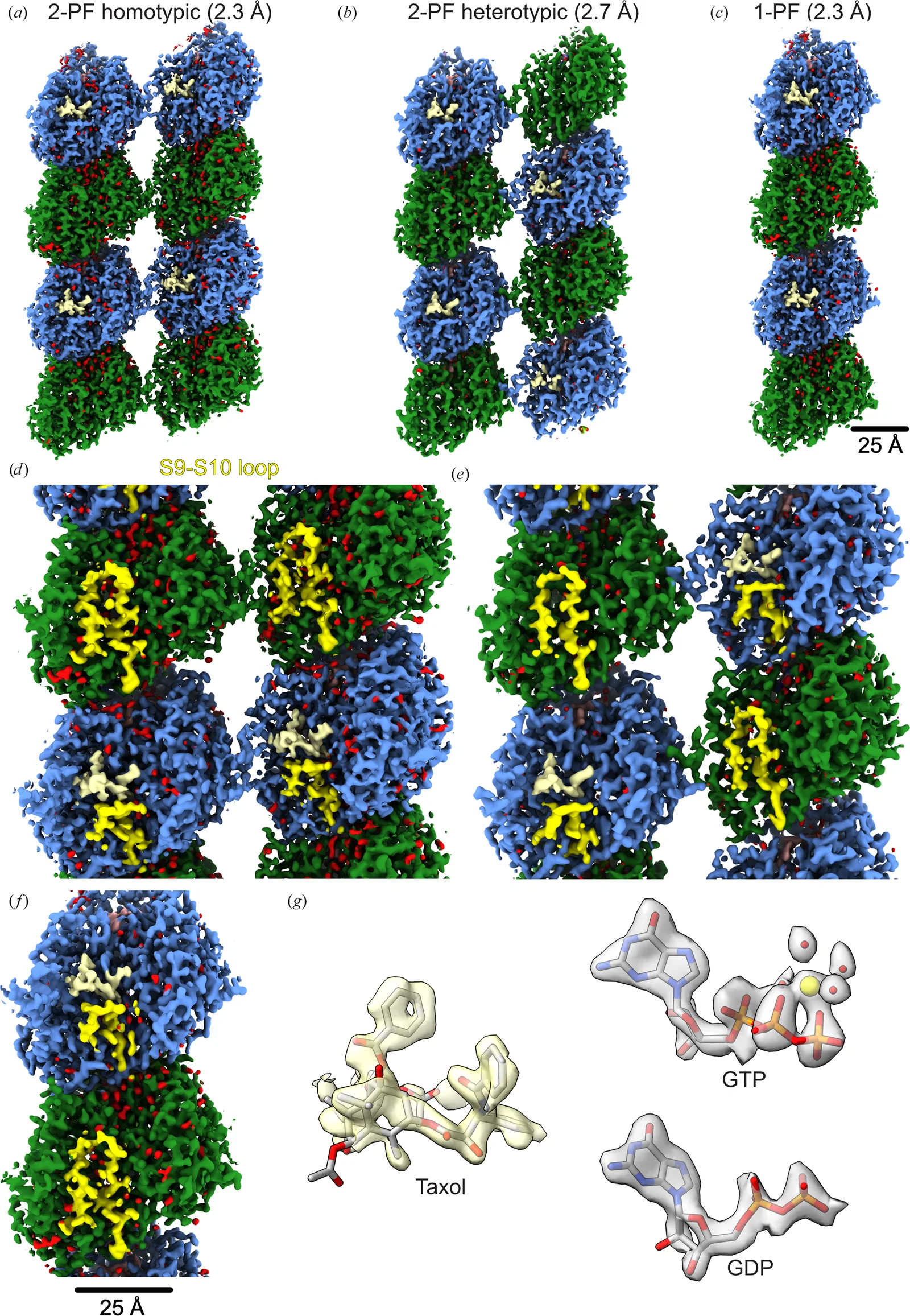
Processing of undecorated Taxol-bound human MTs using the *CsMT* workflow. (*a*) 2-PF homotypic (B-lattice) map at 2.3 Å resolution (2.2 Å using density modification; EMDB entry EMD-78103). (*b*) 2-PF heterotypic (A-lattice) map at 2.7 Å resolution (2.6 Å using density modification; EMDB entry EMD-78104). (*c*) 1-PF map at 2.3 Å resolution (2.2 Å using density modification; EMDB entry EMD-78112). Maps displayed were post-processed using *Phenix* density modification. Color: α-tubulin, green; β-tubulin, light blue; Taxol, beige; water, red; (*d*, *e*, *f*) Magnified views of 2-PF homotypic (*d*), 2-PF heterotypic (*e*) and 1-PF (*f*) maps show distinct densities for S9–S10 loops, a feature that distinguishes α- and β-tubulin. (*g*) Densities of Taxol, GDP and GTP from the 1-PF map. The GTP density shows magnesium densities (yellow) and water densities (red).

Applying this workflow to the cryo-EM data of Taxol-stabilized human MTs (Vangos *et al.*, 2026 ▸), we obtained 2.3 Å resolution for single PF reconstructions and 2.3 and 2.7 Å resolution homotypic and heterotypic 2-PF maps, respectively (Figs. 4 ▸*a*–4 ▸*c*, Supplementary Fig. S2 and Supplementary Table S1). The resolutions of the maps are comparable to those of structures obtained using a novel PF-classification-based *RELION* workflow (Vangos *et al.*, 2026 ▸).

When running our map through density modification (Terwilliger *et al.*, 2020 ▸), the resolutions for our single-PF and 2-PF maps are improved to 2.2 Å. All of our maps clearly show the S9–S10 loop and density for the GTP and GDP and Taxol of α-tubulin and β-tubulin (Figs. 4 ▸*d*, 4 ▸*e* and 4 ▸*f*, Supplementary Fig. S3). As a testament to our high-resolution map, we can visualize the Taxol density in our map with unprecedented clarity (Fig. 4 ▸*g*).

Our 2-PF seam map is the best resolved seam reported so far (Fig. 4 ▸*e*). This is a clear indication that our method works well to resolve the seam structure. Using intersection action from Particle Set Tools from *CryoSPARC* in combination with our novel PF-pair classification, we can effortlessly map particle UID for seam reconstruction without any custom scripts for seam search or seam mapping.

### 3.2. *CsMT* can flexibly accommodate decorated/MAP-bound MTs

**Figure 5:**
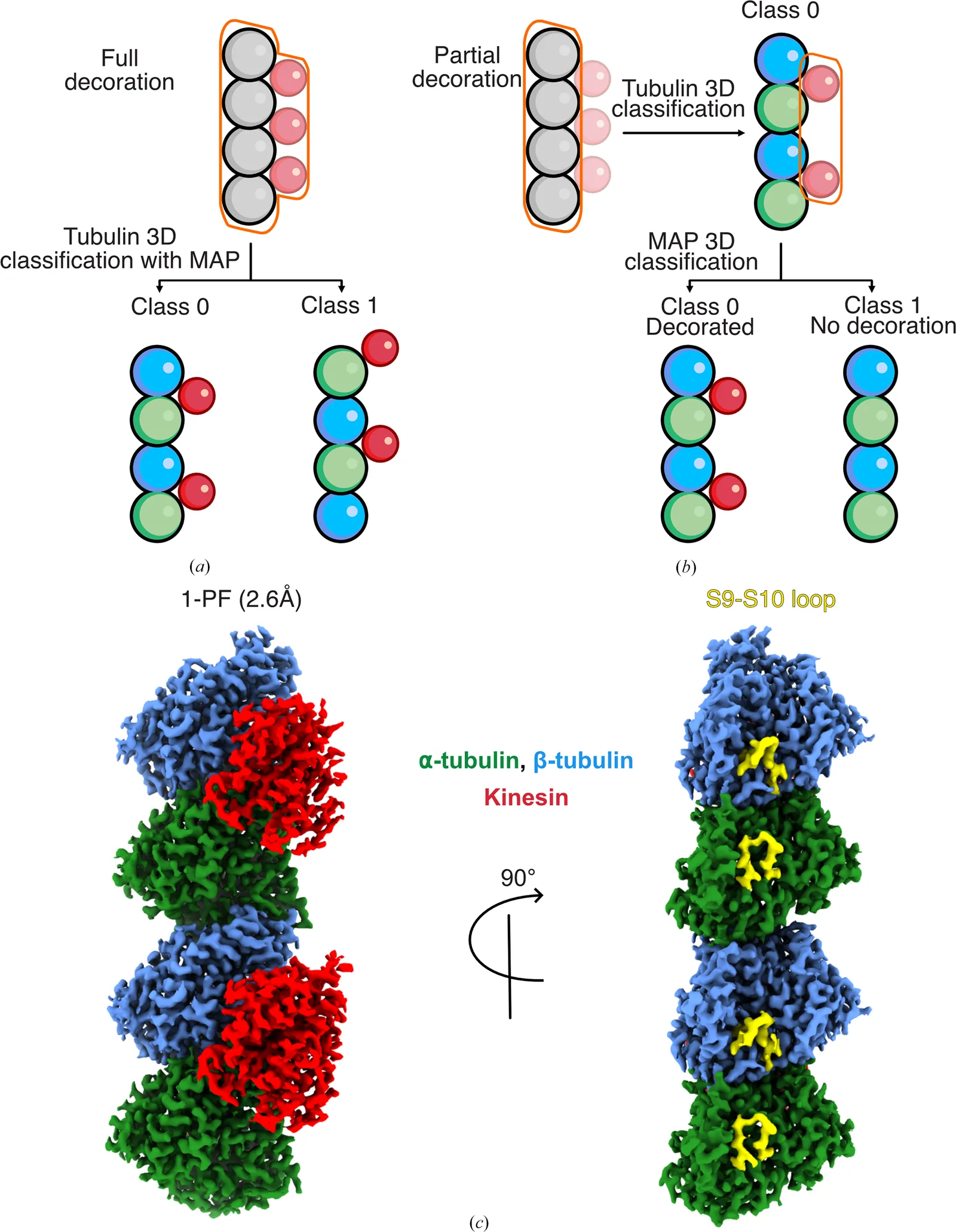
The *CsMT* workflow for decorated MT. (*a*) If the MT is fully decorated, the tubulin classification step in *CsMT* from the α/β-averaged map can also include the decorated MAP by making the classification mask also contain the 4 nm averaged MAP density. (*b*) If the MT is partially decorated, the tubulin classification step in *CsMT* is performed strictly based on the tubulin mask and the synthetic references of α-first and β-first PF. After that, the correctly 8 nm registered PF map is subjected to another classification to separate the decorated PF particles from the undecorated PF particles. (*c*) PF map of tubulin decorated with KIF5A, EMDB entry EMD-78245, reconstructed from EMPIAR-13372 (Zhang *et al.*, 2026 ▸) at 2.6 Å resolution.

Although our workflow is designed for undecorated MTs, simple modifications can be performed to accommodate either fully or partially decorated MTs. For fully decorated MTs, we can perform 3D classification to obtain the correct α/β-tubulin registry at the same time as the MAP classification using a 4 nm repeating mask also containing the MAP densities (Fig. 5 ▸*a*). For partially decorated MTs, we should process 3D classification for the correct α/β-tubulin registry like the undecorated workflow to obtain the correct α/β-tubulin registry map. We can then use a mask for 3D focus classification on the MAP density to separate decorated and non-decorated particles (Fig. 5 ▸*b*). In the specific case where a MAP binds indiscriminately to α- and β-tubulin, the partial decoration workflow can still be used to obtain the density map of MAPs bound to the tubulin dimer.

We applied our workflow to data of MTs decorated with kinesin head domains (EMPIAR-13372; Zhang *et al.*, 2026 ▸). We obtained a map at 2.6 Å resolution which clearly shows the α/β-tubulin and kinesin density (Fig. 5 ▸*c*, Supplementary Fig. S4). Our resolution is slightly better than the reported resolution of 2.8 Å using *MiCSPARC* (Zhang *et al.*, 2026 ▸). This demonstrates that the *CsMT* workflow is also efficient for decorated MTs.

### 3.3. The *CsMT* workflow allows optimization for high-resolution reconstruction

With our efficient *CsMT* workflow and current throughput of data collection (5000–10 000 movies per day), we expect that we can routinely achieve reconstruction at ∼2.5 Å resolution. To obtain high-resolution reconstruction of MTs, there are various factors for consideration. Since the *CsMT* workflow is very simple to implement, we were able to test various parameters to choose the optimum for our data in terms of high resolution and processing time.

#### 3.3.1. Defocus and box-size optimization

High-defocus data inherently limit high-resolution reconstruction due to CTF signal delocalization. For a 300 kV microscope and a 600 Å box size, achieving a 2.5 Å target resolution requires limiting defocus to less than 2.2 µm. This restriction constrains spatial delocalization Δ*r* = λ · Δ*z*/*d* (λ, electron wavelength; Δ*z*, defocus, *d*, resolution) to a maximum of 175 Å, matching the padding threshold outside the MT walls (Rosenthal & Henderson, 2003 ▸). Comparable delocalization constraints apply when recentering and cropping the 2-PF particles. Consequently, a conservative defocus threshold of less than 2.0 µm was enforced to maximize high-resolution signal retention.

#### 3.3.2. Filament-picking optimization

Although most of the pipeline is automated (Fig. 2 ▸), the template-based filament-picking and inspection stages require careful manual tuning, as optimal parameters vary between datasets. Initial optimization should be performed on a subset of approximately 50 micrographs by systematically testing the low and high hysteresis thresholds in the Filament Tracer job, alongside the Normalized Cross-Correlation power and score thresholds in the Inspect Picks step.

In high-magnification datasets such as EMPIAR-13372, there is a significant under-pick of particles due to short filaments and the large box size required. In this case, a filament-extrapolation method such as that introduced by *MiCSPARC* (Zhang *et al.*, 2026 ▸) can nicely improve the particle picking. As shown in this work, spending time to optimize the filament picking can achieve good particle-picking coverage. Therefore, external scripts are best reserved for advanced users, as custom scripting interrupts the seamless *CryoSPARC* workflow.

#### 3.3.3. Heterorefinement optimization

This step is crucial for the success of the workflow. However, it is not always easy to stably run this step as the minor classes such as 11-PF, 12-PF, 15-PF and 16-PF can be as low as 1% of the particles. This leads to attraction problems when larger and better resolved classes attract all the particles from other minor classes. For *CryoSPARC*, restricting the initial resolution to 15 Å and the alignment resolution to 15 Å and turning on Force Hard Classification helps significantly with the convergence of the PF sorting. Additionally, limiting the reference framework to 12-PF through 15-PF proves to be beneficial; the 12-PF classification absorbs neighboring 11-PF particles, whereas the 15-PF class similarly assimilates 16-PF particles. However, the sorting will still struggle with small datasets when the particles from minor classes will not yield a ∼15 Å map. In this case, perhaps limiting the references only to the major classes (13-PF and 14-PF) would be more appropriate.

#### 3.3.4. Mask-size optimization

For PF classification, focused masks containing two tubulin dimers per PF (∼400 kDa molecular weight) provided sufficient signal for accurate alignment, performing comparably to larger three-dimer masks (∼600 kDa). During the final 2-PF local refinements, the smaller two-dimer × 2-PF mask yielded the same resolutions: 2.3 Å for the homotypic B-lattice and 2.70 Å for the heterotypic seam.

#### 3.3.5. Centering, cropping and computational efficiency

Centering and cropping the 2-PF density based on the focused refinement mask is critical for both computational performance and rotational accuracy. Centering places the fulcrum of the rotational search directly at the center of mass of the focused mask, improving alignment precision. Structurally, cropping the particles from a 720 × 720 pixel box down to a 400 × 400 pixel box reduced the storage footprint of a 2.6-million particle dataset from ∼2.4 TB to ∼770 GB (a 68% reduction). This significantly optimized data management; for a subset of one million particles, SSD caching took only 2 h over our network and the subsequent local refinement was completed in 4 h on a single NVIDIA RTX 3090 GPU.

#### 3.3.6. Classification strategy

Unlike *RELION*, *CryoSPARC* does not feature a regularization parameter (T-value) to tune 3D classification sensitivity. To accurately resolve the expected ∼50:50 distribution between the ‘α-first’ and ‘β-first’ classes, specific classification parameters must be enforced. Activating Force Hard Classification prevents particles from weights-blending across classes, while setting the O-EM learning rate to 0 and enforcing Filter to Resolution at 4 Å ensures that the initial synthetic references strictly guide the Full-EM classification phase without drifting.

#### 3.3.7. Gold-standard Fourier shell correlation (FSC) validation

Due to particle overlap along the helical axis, filament reconstructions are highly susceptible to resolution inflation caused by duplicate or overlapping particles leaking into both half-datasets during refinement. This explains the fact that certain MT maps appear worse than the reported resolution. To prevent this, particles belonging to the same physical filament must be restricted to the same half-set for gold-standard processing.

While maintaining filament IDs across software suites can be problematic due to data loss during conversion, our workflow mitigates this by enforcing a 4 nm picking interval and 4 nm helical symmetry during refinement. However, because filament-ID-based splitting can still be imperfect, partitioning the gold-standard split strictly by micrograph ID (a feature native to *CryoSPARC*) provides the most rigorous and accurate method for FSC resolution estimation in large datasets. In addition, map-to-model FSC offers a robust, quantitative method to evaluate model accuracy and prevent over-fitting against the experimental map (Afonine *et al.*, 2018 ▸).

## 4. Discussion

In this work, we developed *CsMT*, a *CryoSPARC* workflow to reconstruct MTs. Notably, our new processing workflow, available as a single import-ready JSON file (https://github.com/builab/CsMT), achieved 2.3 Å global resolution for a single-PF map of MTs in approximately 157 h using one GPU processing of ∼3200 movies (Supplementary Table S2). Compared with the seam-search approach, our approach does not depend on picking a long filament. Thus, we do not need manual picking (Cook *et al.*, 2020 ▸) or multiple scripts for filament interpolation, angle unification or seam smoothing after classification such as in *MiCSPARC* (Zhang *et al.*, 2026 ▸).

Furthermore, our analysis suggests that there is not a single seam along the MT. The number of particles of seam versus B-lattice is about 1:4 for our 13-PF MTs, instead of a 1:12 ratio if a single-seam assumption is true. Our observation is supported by good resolution obtained from our seam map, which suggests the majority of the classification is correct as otherwise 50% of the particles would be assigned wrongly in our seam particle set based on a one-seam assumption. A cryo-electron tomography study using kinesin-decorated MTs visualizes multiple seams (Guyomar *et al.*, 2022 ▸), supporting our observation. On the other hand, the seam-search approach still works well. It suggests that *in vitro* reconstituted MTs likely have a main seam running along them but might contain other seams due to imperfect polymerization.

The single-PF approach likely yields better resolution than the 2-PF approach as it is less impacted by biological flexibility and the flattening of MTs during vitrification. On the other hand, the PF-pair approach allows a nice visualization of the seam or the B-lattice instead of a single PF. This is probably a big advantage if the MAP of interest is binding between two PFs, such as EB proteins binding to between four tubulin dimers (Maurer *et al.*, 2012 ▸) or SPEF1 binding between the seam (Legal *et al.*, 2025 ▸).

The seam-search approach allows reconstruction of the full MT (Zhang & Nogales, 2015 ▸; Zhang *et al.*, 2026 ▸). However, reconstructing the full MT can be achieved efficiently in *ChimeraX* by creating a composite map from multiple copies of the 2-PF maps in *ChimeraX* using the ‘volume maximum’ command (Legal *et al.*, 2025 ▸; Meng *et al.*, 2023 ▸). Furthermore, it is also possible to use scripts to analyze distorted MTs or lattice defects, as demonstrated previously (Debs *et al.*, 2020 ▸).

In the future, a single-workflow *CsMT* might be able to be tweaked for fully autonomous analysis. All of the references and masks can be re-used if we use the same magnification. Selection of 2D classes can be performed automatically in a large dataset with a resolution threshold or using a tool such as *CryoSIFT* (Schäfer *et al.*, 2025 ▸). The only step that requires manual optimization is filament picking and inspection. In a large dataset, it is possible to use stringent criteria, and we are still able to pick enough particles for reconstruction.

As cryo-EM technology continues to evolve, achieving sub-2 Å resolution for MTs is becoming increasingly feasible using advanced detectors and larger datasets. Our *CsMT* framework contributes to the standardization of the MT reconstruction process, enabling researchers to prioritize sample optimization and data quality to better elucidate MT structure and function.

## Supplementary Material

EMDB reference: Taxol-stabilized human microtubule, 2-protofilament homotypic map, EMD-78103

EMDB reference: 2-protofilament heterotypic map, EMD-78104

EMDB reference: 1-protofilament map, EMD-78112

EMDB reference: KIF5A-decorated microtubule, 1-protofilament map, EMD-78245

Supplementary Figures and Tables. DOI: 10.1107/S2059798326010144/sor5009sup1.pdf

## Data Availability

The *CryoSPARC* workflow JSON file is available at https://github.com/builab/CsMT. Data and scripts to plot MT resolution histogram and software usage are also available from the GitHub page. EM maps of 2-PF homotypic, 2-PF heterotypic and 1-PF of Taxol-stabilized human MT, and 1-PF of MT with KIF5A have been deposited in the Electron Microscopy Data Bank (EMDB) under accession numbers EMD-78103, EMD-78104, EMD-78112 and EMD-78245.
