# Supplementary material for: CsMT: a robust and streamlined CryoSPARC workflow for cryo-EM reconstruction of microtubules
Source: Acta Crystallogr D Struct Biol. 2026 Oct 14;82(Pt 11):1212–23. doi: 10.1107/S2059798326010144 (PMC13717834; doi:10.1107/S2059798326010144)
Supplement: Supplementary file 1 [file d-82-01212-sup1.pdf]

Supplemental Tables

Supplemental Table 1: Cryo-EM data collection and processing

	**Undecorated**			**Decorated**
**EMPIAR**	[23]			EMPIAR-13372
**Reconstruction**	2-PF homotypic	2-PF heterotypic	1-PF	1-PF
**Microscope**	Titan Krios G3			Titan Krios
**Voltage (keV)**	300			300
**Detector**	K3 BioQuantum			K2 Summit
**Magnification**	105,000 X			78,125 X
**GIF slit width**	20 eV			20 eV
**Number of movies**	3,197			1,676
**Number of frames**	50			62
**Exposure time (s)**	12			13
**Total dose (e/Å²)**	50			65
**Original pixel size (Å/pixel)**	0.834			0.64
**Processing pixel size**	0.834	0.834	0.834	0.835
**Initial number of particles**	220,251	220,251	220,251	19,888
**Symmetry imposed**	C1	C1	C1	C1
**Final number of particles**	997,466	265,821	1,275,502	273,875
**Final processing box size**	400	400	400	400
**Map resolution (Å)**	2.3	2.7	2.3	2.6
**Model building**	Rigid body fit	Rigid body fit	Rigid body fit	Rigid body fit
**Accession number**	EMD-78103	EMD-78104	EMD-78112	EMD-78245

Supplemental Table 2: Cryo-EM data processing time for undecorated MT. * denoted steps affected heavily by data read/write speed in our cluster system.

Step	Number of GPU	Running Time (hours)
Patch Motion Correction	1	20
Patch CTF Estimation	1	6
Filament Tracer (templates generation)	1	0.7
Inspect Picks (templates generation)	0	0.01
Extraction (templates generation)	1	0.3
2D Classification (templates generation)	2	0.2
Select 2D Classes	0	0.01
Filament Tracer (with templates)	1	2.2
Inspect Picks (with templates)	0	0
Extraction (with templates)	1	1.25
2D Classification (first round)	2	0.5
Select 2D Classes	0	0.01
2D Classification (second round)	2	1
Select 2D Classes	0	0.01
Hetero. Refine.	1	0.7
Extract Mics of MT Class	1	1.5
Helix Refine	1	2.7
Local CTF Refinement	1	0.15
Global CTF Refinement	1	0.13
Helix Refine	1	2.77
Reference based MC	2	9.33
Helix Refine	1	3.66
Symmetry Expansion	0	0.02
Local Refine	1	14
Particle Subtraction*	1	36
Downsample Particles*	0	32
Volume Alignment Tool	1	0.13
Local Refine PF*	1	10
3D Classification	1	5.5
Local Refine (8-nm PF)	1	2.5
Local CTF Refinement (8-nm PF)	1	0.25
Final Local Refine (8-nm PF)	1	3

Supplemental Figures

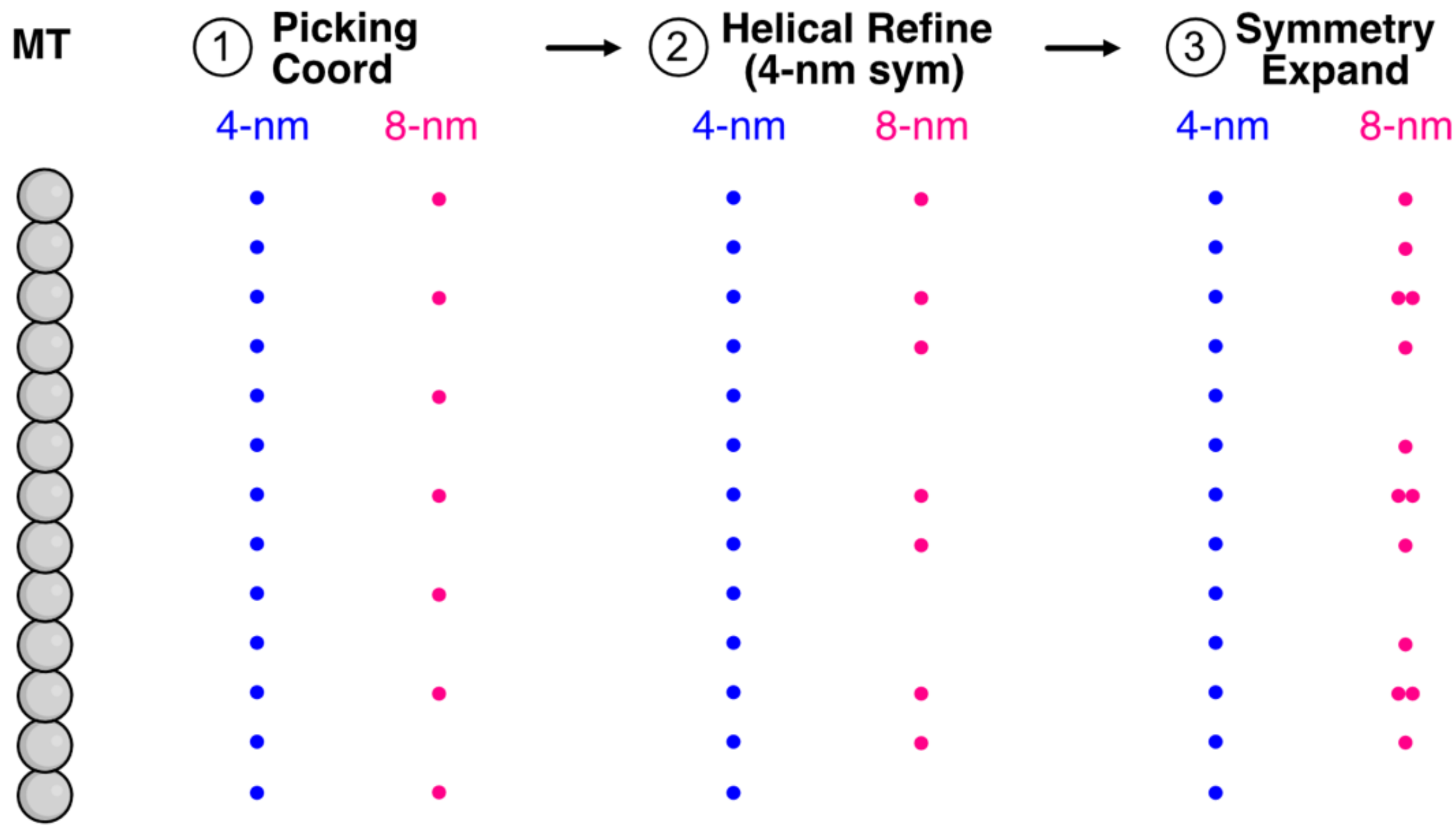

Supplementary Fig. 1: 4-nm picking avoids particle duplication during processing. Step 1 represents the picking coordinates along a microtubule with 4-nm or 8-nm picking. During helical refinement with 4-nm symmetrization (Step 2), the 8-nm picked particles might shift along the filament axis while the 4-nm picked particles are constrained to not shift along the filament axis, imposed by the helical refinement. Symmetry expansion of the particles after refinement will lead to more duplicate particles in the case of 8-nm pick particles.

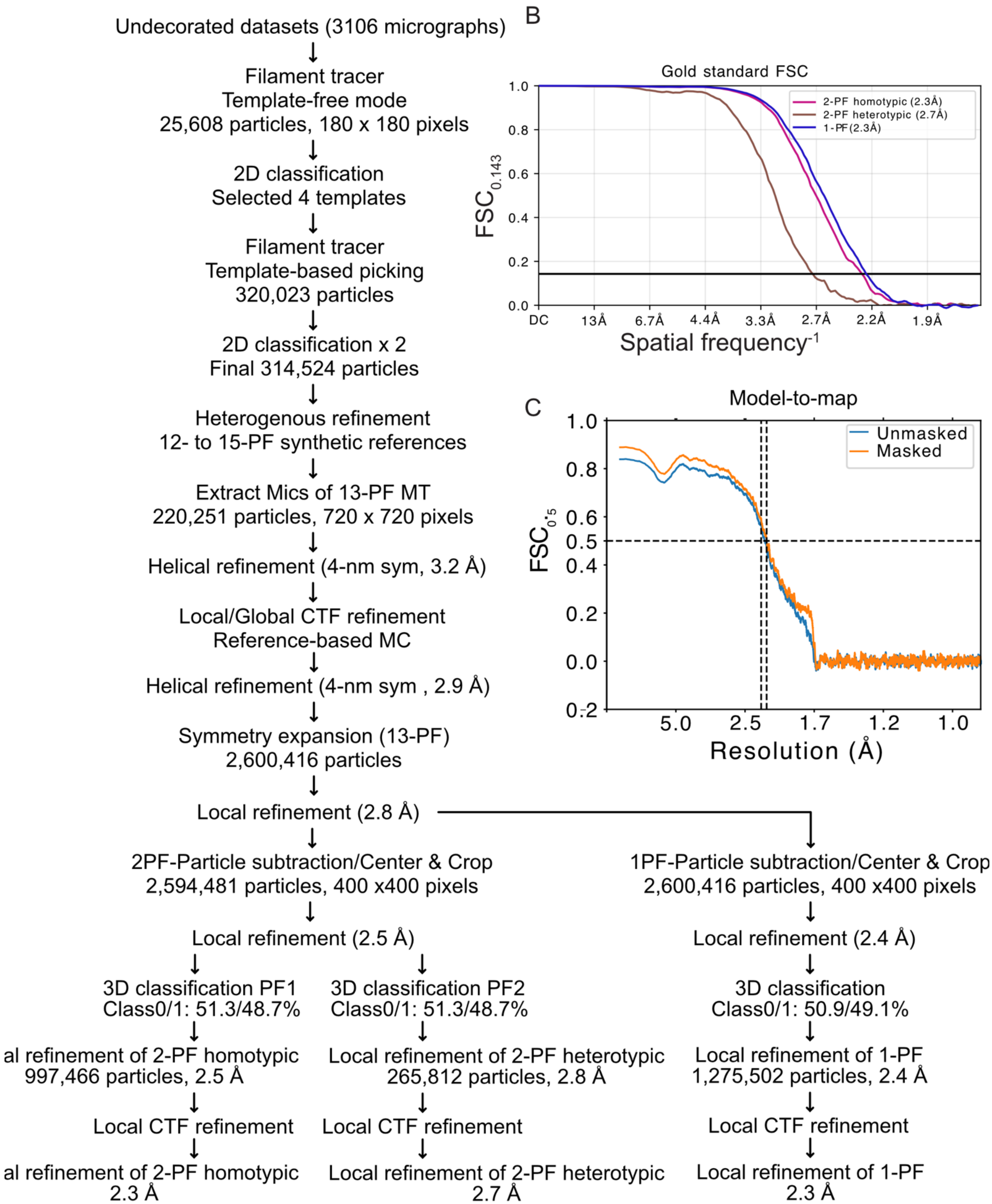

Supplementary Fig. 2: Processing undecorated data of taxol stabilized human MT. [23] (A) Processing workflow of undecorated data. (B) Fourier shell correlation (FSC) of the 2-PF heterotypic, 2-PF homotypic and 1-PF map. (C) Model-to-map FSC calculated using Phenix for the 2-PF homotypic map indicates a 2.2 Å resolution.

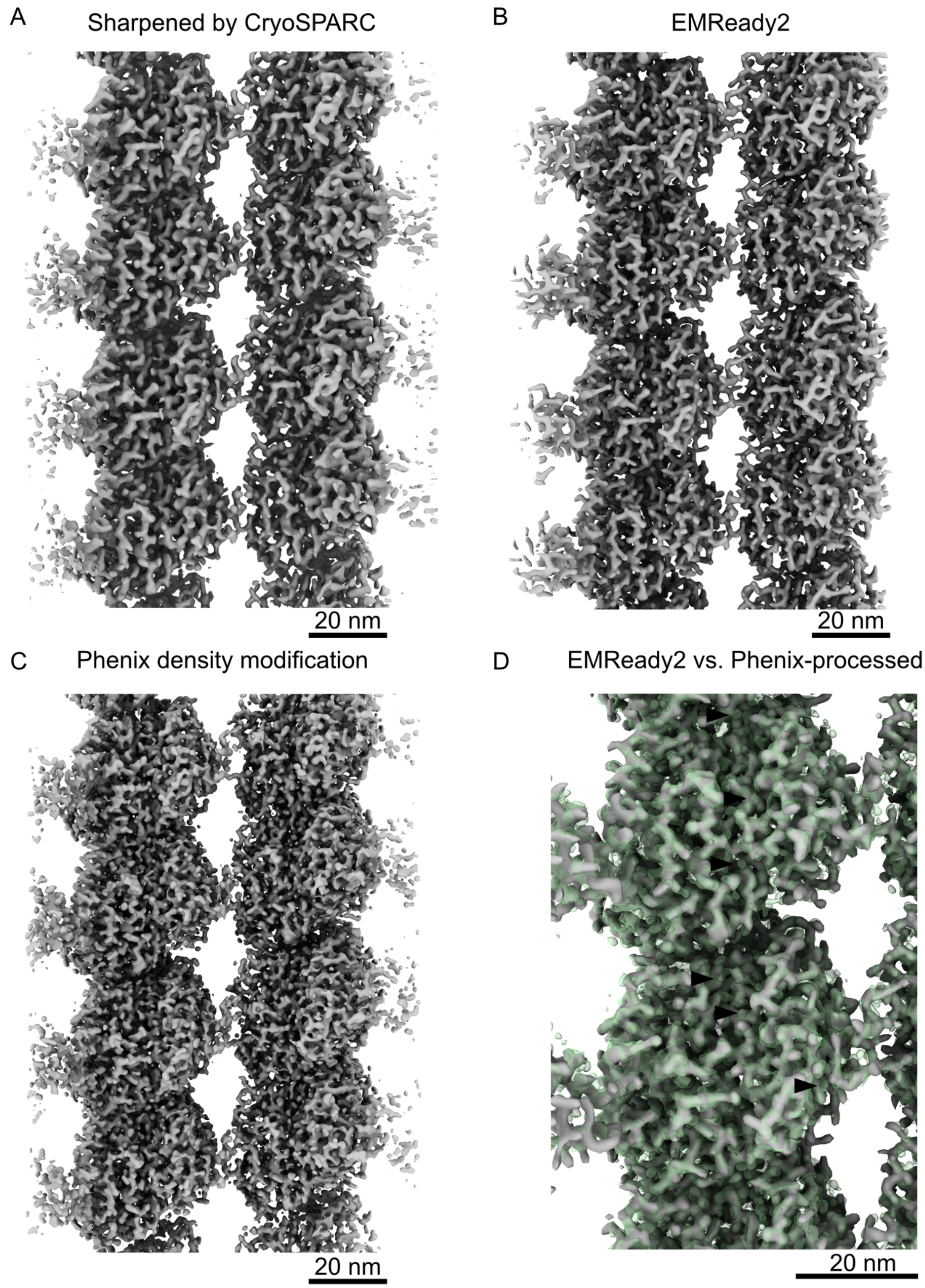

Supplementary Fig. 3: Comparison of (A) global B-factor sharped maps by CryoSPARC, (B) EMReady2-processed map and (C) Phenix density modified map and of the 2-PF heterotypic map. (D) Superimposed of Phenix density modified map (transparent mint green) vs. the EMRead2-processed map (gray). Water molecules are visible in the Phenix density-modified map (indicated by black arrows) but are absent in the EMReady2-processed map.

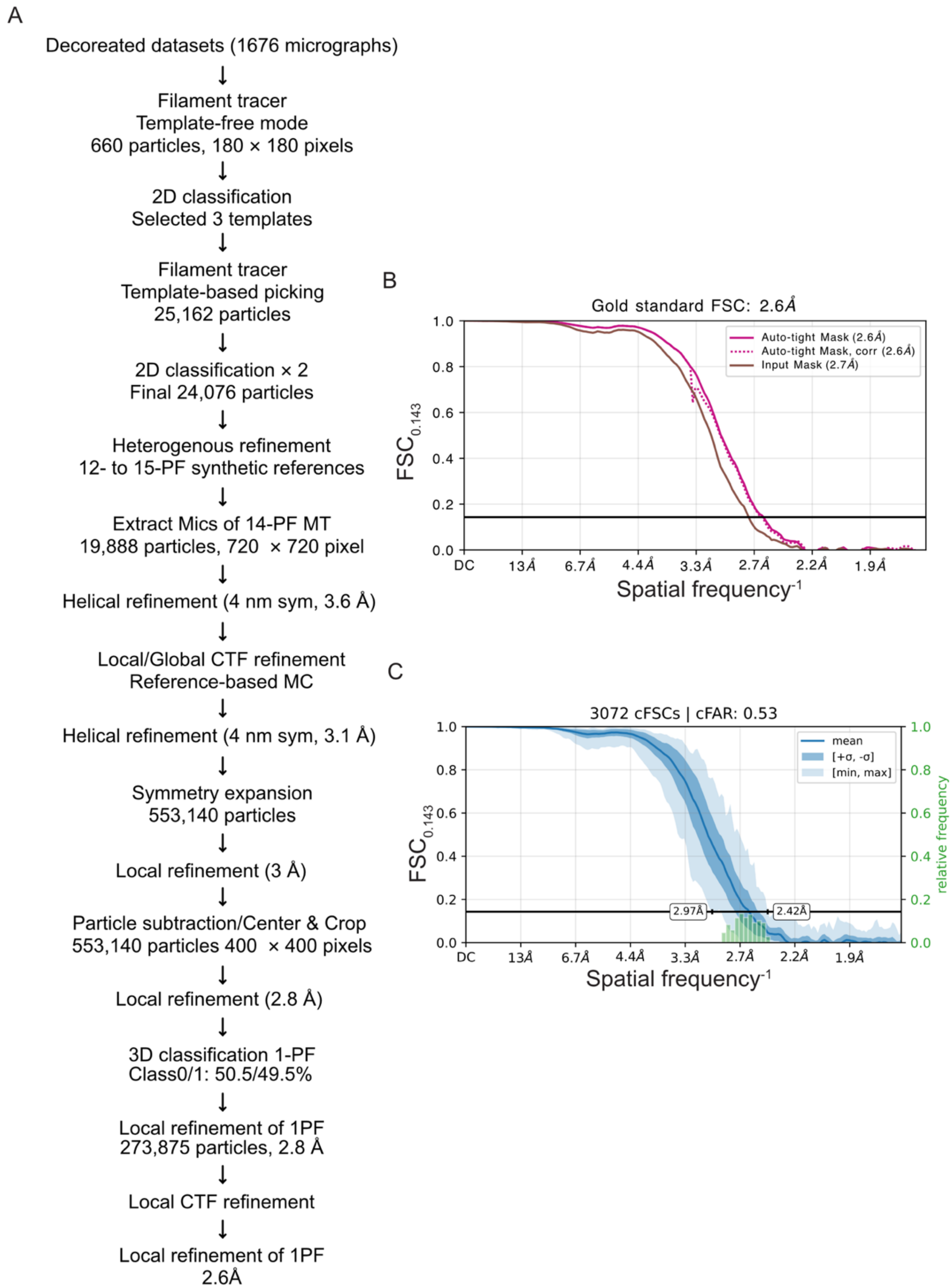

Supplementary Fig. 4: Processing of cryo-EM dataset EMPIAR-13372 [20]**.** (A) Processing workflow of MT decorated with KIF5A (EMPIAR-13372). (B) Fourier shell correlation (FSC) of 1-PF reconstruction obtained in A. (C) Conical Fourier Shell Correlation (cFSC) of the 1-PF reconstruction obtained in A.
